# Evaluation of the performances of six commercial kits designed for dengue NS1 and anti-dengue IgM, IgG and IgA detection in urine and saliva clinical specimens

**DOI:** 10.1186/s12879-016-1551-x

**Published:** 2016-05-16

**Authors:** Anne-Claire Andries, Veasna Duong, Sivuth Ong, Sopheaktra Ros, Anavaj Sakuntabhai, Paul Horwood, Philippe Dussart, Philippe Buchy

**Affiliations:** Institut Pasteur in Cambodia, International Network of Pasteur Institutes, Virology Unit, Phnom Penh, Cambodia; Institut Pasteur, Functional Genetics of Infectious Diseases Unit, Paris, France; Centre National de la Recherche Scientifique, Unité de Recherche Associée 3012, Paris, France; GlaxoSmithKline, Vaccines R&D, Singapore, Singapore

**Keywords:** Dengue, Diagnostic, Rapid diagnostic test, Saliva, Urine, NS1, Antibodies

## Abstract

**Background:**

Rapid diagnostic tests (RDTs) have been commercialized in order to help physicians in dengue diagnosis. Until recently, only blood samples were used for those tests but it has been shown in several studies that urine and saliva can also be employed for dengue diagnosis. RDTs for the detection of NS1 antigen and anti-dengue IgG, IgM and IgA in urine and saliva specimens have thus been developed by Standard Diagnostics. The aim of this study was to evaluate the performances these new commercial assays.

**Methods:**

Two panels of clinical specimens were used: one for the evaluation of the NS1-detection devices and the second for the evaluation of the antibody-detection kits. Each panel consisted of urine and saliva specimens collected sequentially from 86 patients with a confirmed dengue infection. A total of 291 saliva and 440 urine samples were included in the NS1-evaluation panel and 530 saliva and 528 urine specimens constituted the antibody-evaluation panel. All samples were tested in parallel by in-house ELISAs and by the commercial RDTs.

**Results:**

The RDTs demonstrated an overall sensitivity of 15.5 %/27.9 %/10.7 % for NS1/IgG/IgA detection in urine samples and 20.4 %/ 34.8 %/11 %/6.2 % for NS1/IgG/IgM/IgA detection in saliva samples. Compared to the in-house NS1 ELISA, the results obtained with the NS1 RDT demonstrated a good correlation with urine samples (kappa coefficient: 0.88) but not with saliva specimens (kappa coefficient: 0.28). RDTs designed for antibody detection in saliva and urine were extremely specific (100 %), but less sensitive than the in-house ELISAs (i.e., reduction of the overall sensitivity by 12.2 % for the RDT designed for IgG detection in urine and by 23.7 % for the RDT detecting anti-DENV IgM in saliva). IgM were not detected in urine, either by RDT or ELISA*.*

**Conclusions:**

Although the RDTs evaluated here offer an apparently attractive approach for dengue diagnosis, this study suggests that these new commercial kits would require further improvement to increase the sensitivity.

**Electronic supplementary material:**

The online version of this article (doi:10.1186/s12879-016-1551-x) contains supplementary material, which is available to authorized users.

## Background

Dengue is the most common arboviral disease worldwide, with almost 130 countries of tropical or subtropical regions for which there is good evidence of dengue occurrence [1]. Dengue illness begins with undifferentiated symptoms, common to several other infectious diseases such as malaria, influenza or other arboviral diseases. After a few days, most patients begin to recover but some progress to severe hemorrhagic disease forms that can result in death without proper medical care. Early clinical management based on accurate rehydration can prevent life-threatening complications [2]. Hence, the need for an early diagnosis of dengue infection.

Direct diagnosis of dengue is based on dengue virus (DENV) isolation, or detection of the viral genome or NS1 antigen. Indirect diagnosis using serological methods to detect anti-DENV IgM and IgG is commonly employed. Anti-DENV IgA are also detectable during a DENV infection but tests to detect this immunoglobulin isotype are not often used in routine diagnosis [3–5]. Rapid diagnostic tests (RDTs) for NS1 and anti-DENV antibody detection in blood have been developed in order to help physicians make rapid and informed decisions about adapted clinical management. Until recently, only blood samples were used for dengue diagnosis but it has been shown in several studies that urine and saliva can also be employed [6–11].

Standard Diagnostics (SD; Kyonggi-do, South Korea) has developed RDTs able to detect markers of dengue infection in urine and saliva specimens. They designed a NS1 RDT, a combined anti-DENV IgM/IgG RDT and an anti-DENV IgA RDT adapted for testing both urine and saliva clinical specimens. The aim of this study was to evaluate the performances of these diagnostic tools. The results obtained with the RDTs were compared with those of in-house ELISAs.

## Methods

### Clinical samples

Two panels of clinical specimens were used: the first one for the evaluation of the NS1 test and the second for the antibody detection assays. Each panel consisted of urine, saliva and plasma specimens collected sequentially from 86 patients with a confirmed dengue infection. A confirmed dengue case was defined by the detection of viral RNA by RT-PCR and/or the detection of the NS1 protein and/or an IgM seroconversion and/or a fourfold antibody titer increase measured by hemagglutination inhibition assay (HIA) in paired plasma of patients presenting with symptoms suggestive of a dengue infection. Fifty patients presented with mild symptoms defined by the 1997 WHO criteria as classical dengue fever (DF) and 36 patients experienced severe symptoms compatible with the diagnostic of dengue hemorrhagic fever (DHF) or dengue shock syndrome (DSS) [12]. The immune status of 73 patients was determined by HIA: 24 patients experienced a primary infection whereas 49 had a secondary infection. The NS1 panel consisted of samples collected during hospitalization (i.e., from day 1 to 12 after onset of fever), whereas samples collected during a weekly follow-up until three months after discharge were also included in the second panel designed for the evaluation of the antibody-detection RDTs. Four to ten urine and saliva specimens and two to five plasma samples obtained from each patient were included in these panels. A total of 241 plasma, 291 saliva and 440 urine samples were included in the NS1-evaluation panel and 292 plasma, 530 saliva and 528 urine specimens constituted the antibody-evaluation panel. The samples of the antibody panel were classified into three categories based on whether the corresponding plasma sample collected at the same time point was weakly, averagely or highly positive in antibody based on the optical density (OD) measured by ELISA. Similarly, the samples included in the NS1 panel were classified into weakly, averagely or highly positive based on the concentration of NS1 measured by ELISA in the corresponding plasma specimen. The NS1 quantification was only performed for samples obtained from patients infected by a virus from serotype 1 (DENV-1) as only quantified recombinant NS1 protein from DENV-1 was available in our laboratory. The three categories were defined arbitrarily by calculating the 1^st^ and 3^rd^ quartile of 300 OD/concentration values of antibodies and NS1 protein obtained with 300 positive plasma samples. Weakly, averagely and highly positive plasma samples corresponded to samples with a result lower than the 1^st^ quartile, between the 1^st^ and the 3^rd^ quartile and greater than the 3^rd^ quartile, respectively. Twenty-five specimens obtained from healthy controls recruited during a community-based study were also added to the panels in order to assess the specificity. These negative controls were obtained from household members of some of the patients identified during the hospital-based study and who did not experience any symptoms and had no biological evidence of DENV infection (DENV RT-PCR negative, NS1 RDT negative and absence of HI antibody).

### In-house ELISAs

The RDTs evaluated in this study were compared to in-house ELISAs and we will refer to them as “Institut Pasteur Cambodia (IPC) ELISAs” throughout the manuscript. A capture ELISA was used to detect NS1 in plasma, urine and saliva. Anti-DENV IgG were detected in plasma, urine and saliva by an indirect ELISA whereas anti-DENV IgM and IgA were detected by capture ELISAs (MAC-ELISA and AAC-ELISA, respectively). These ELISAs and their performances were previously described in detail by Andries et al. [11].

### Rapid diagnostic tests

In this study, prototype immunochromatographic kits developed by Standard Diagnostics for NS1 and anti-DENV IgG, IgM and IgA detection were evaluated. Anti-DENV IgG and IgM were detected by the same device, whereas NS1 and anti-DENV IgA were detected by two other distinct devices. Each kit was either designed for saliva or urine specimen analysis. The characteristics of the six different devices are summarized in the Additional file 1. All tests were strictly performed according to the manufacturer’s instructions provided with the kits.

### Statistical analysis

Statistical analysis was performed using STATA version 11.0 (StataCorp, USA). Significance was assigned at *P* < 0.05 for all parameters and were two-sided. Uncertainty was expressed by 95 % confidence intervals (CI95). Statistical differences between various categorical groups were detected using McNemar’s test. Agreement between IPC ELISAs and RDTs was measured by agreement percentage and kappa coefficient.

## Results

### DENV NS1 RDTs

A specificity of 100 % (25/25) was obtained for the NS1 RDT in urine, whereas the specificity of the saliva-based RDT was 84 % (21/25).

The RDT and the IPC ELISA detected NS1 in 15.5 % (68/440, CI95 = [12.2–19.2]) and 14.3 % (63/440, CI95 = [11.2–17.9]) of the urine samples, respectively. The agreement between the two urine-based tests was 97.1 % with a kappa coefficient of 0.88 (Additional file 2a). The NS1 RDT and ELISA tested positive in 20.4 % (59/289, CI95 = [16.2–25.9]) and 24.7 % (72/291, CI95 = [19.9–30.1]) of the saliva specimens, respectively. An agreement of 74.0 % was obtained between both tests with a kappa coefficient of 0.28 (Additional file 2b). Two saliva samples were excluded because an invalid result (no control line) was obtained with the RDT. In total, 66 % (159/241, CI95 = [59.6–71.9]) of the plasma samples collected at the same time points from the same dengue-confirmed patients as the urine and saliva specimens tested positive by NS1 IPC ELISA.

The NS1 detection rate by ELISAs and RDTs was systematically higher in urine and saliva samples obtained from patients with high NS1 concentration in the plasma specimens obtained at the same time points (Table 1).

**Table 1:** Detection rate of NS1 in urine and saliva samples

	Urine		Saliva	
	IPC NS1 capture ELISA	NS1 RDT	IPC NS1 capture ELISA	NS1 RDT
Weakly positive plasma	10.3 % (3/29)	10.3 % (3/29)	13.3 % (2/15)	20 % (3/15)
Averagely positive plasma	16.7 % (9/54)	16.7 % (9/54)	48.5 % (16/33)	18.2 % (6/33)
Highly positive plasma	50 % (17/34)	50 % (17/34)	64.7 % (11/17)	47.1 % (8/17)
*p*-value ^a^	<0.001	<0.001	0.011	0.094

The detection rates of NS1 in urine and saliva samples are presented according to the NS1 level in the corresponding plasma samples

^a^ Comparison of NS1 positive rate in urine or saliva from patients whose corresponding plasma tested weakly, averagely or highly positive by NS1 ELISA

The sensitivity of urine- and saliva-based NS1 tests was highest in samples collected 4–7 days after the onset of fever (DAOF) from patients experiencing a primary infection (Table 2). IPC ELISA and RDT demonstrated both a maximal sensitivity for NS1 detection of 21.5 % (17/79, CI95 = [13.1–32.2]) in the urine samples collected during the 4 to 7 first days of the disease in patients experiencing a primary infection. A sensitivity of 46.7 % (28/60, CI95 = [33.7–60]) was observed with the saliva-based IPC ELISA whereas the RDT’s sensitivity only reached a maximum of 33.9 % (20/59). In comparison, 93.9 % (31/33, CI95 = [79.8–99.3]) of the plasma collected at DAOF 4–7 from the same patients experiencing a primary infection tested positive for NS1.

**Table 2:** Sensitivity of the different diagnostic tools for NS1 detection in plasma, urine and saliva

			Total	Primary infection	Secondary infection
Plasma	ELISA	All samples	66 % (159/241)	95.7 % (66/69)	51.1 % (69/135)
		DAOF ≤ 3	94.4 % (67/71)	100 % (29/29)	88.6 % (31/35)
		DAOF 4–7	66.7 % (82/123)	93.9 % (31/33)	51.4 % (36/70)
		DAOF 8–12	21.3 % (10/47)	85.7 % (6/7)	6.7 % (2/30)
Urine	ELISA	All samples	14.3 % (63/440)	18.3 % (23/126)	8.1 % (20/246)
		DAOF ≤ 3	9.9 % (8/81)	15.6 % (5/32)	2.4 % (1/41)
		DAOF 4–7	19.2 % (52/271)	21.5 % (17/79)	12.3 % (19/154)
		DAOF 8–12	3.4 % (3/88)	6.7 % (1/15)	0 % (0/51)
Urine	RDT	All samples	15.5 % (68/440)	18.3 % (23/126)	10.6 % (26/246)
		DAOF ≤ 3	11.1 % (9/81)	15.6 % (5/32)	4.9 % (2/41)
		DAOF 4–7	21 % (57/271)	21.5 % (17/79)	15.6 % (24/154)
		DAOF 8–12	2.3 % (2/88)	6.7 % (1/15)	0 % (0/51)
Saliva	ELISA	All samples	24.7 % (72/291)	43.5 % (37/85)	15.5 % (27/174)
		DAOF ≤ 3	25.5 % (12/47)	41.2 % (7/17)	19.2 % (5/26)
		DAOF 4–7	29.9 % (55/184)	46.7 % (28/60)	19.4 % (21/108)
		DAOF 8–12	8.3 % (5/60)	25 % (2/8)	2.5 % (1/40)
Saliva	RDT	All samples	20.8 % (60/289) ^b^	31 % (26/84) ^a^	16.2 % (28/173) ^a^
		DAOF ≤ 3	14.9 % (7/47)	23.5 % (4/17)	11.5 % (3/26)
		DAOF 4–7	25.7 % (47/183) ^a^	33.9 % (20/59) ^a^	19.4 % (21/108) ^a^
		DAOF 8–12	10.2 % (6/59) ^a^	25 % (2/8)	10.3 % (4/39) ^a^

The sensitivity of the different diagnostic tools for NS1 detection in plasma, urine and saliva of DENV-infected patients is presented according to the time of sampling after the onset of the fever and the immune status of the patients

*DAOF* Day After Onset of Fever

^a^ One invalid result

^b^ Two invalid results

### Anti-DENV antibody RDTs

All 25 urine and 25 saliva samples obtained from the healthy controls tested negative for IgG, IgM and IgA by RDTs.

Anti-DENV IgG were detected in 27.9 % (145/520, CI95 = [24.1–32]) and 40.4 % (210/520, CI95 = [36.1–44.7]) of the urine samples by RDT and IPC ELISA, respectively. An agreement of 86.7 % was obtained between the two assays and the kappa coefficient was 0.71 (Additional file 2c).

A total of 10.7 % (56/522, CI95 = [8.2–13.7]) and 27.2 % (142/522, CI95 = [23.4–31.2]) of the urine samples tested positive by RDT and IPC ELISA for anti-DENV IgA detection. Results were concordant for 81.2 % of the urine specimens with a kappa coefficient of 0.42 (Additional file 2d).

The sensitivity of IgG and IgA antibody detection in urine by the ELISAs was significantly higher than the sensitivity of the RDTs (McNemar *p*-value ≤0.001 for IgG and IgA).

Anti-DENV IgM were not detected in urine specimens, either by RDT or by IPC ELISA.

The overall detection rates of anti-DENV IgG, IgM and IgA in saliva samples were respectively 34.8 % (181/520, CI95 = [30.7–39.1]), 11 % (57/520, CI95 = [8.4–14]) and 6.2 % (32/520, CI95 = [4.2–8.6]) with the RDTs, and 51.5 % (268/520, CI95 = [47.1–55.9]), 34.8 % (181/520, CI95 = [30.7–39.1]) and 22.7 % (118/520, CI95 = [19.2–26.5]) with IPC ELISAs. Percentages of agreement between RDTs and IPC ELISAs for IgG, IgM and IgA detection in saliva were 81 %, 75.8 and 82.3 %, respectively. The corresponding kappa coefficients were 0.62, 0.36 and 0.32 (Additional file 2e, f and g). Saliva-based ELISAs were significantly more sensitive than saliva-based RDTs (McNemar *p*-value ≤0.001 for all antibodies).

In comparison, IPC ELISAs for IgG, IgM and IgA detection in the plasma specimens obtained at the same time point from the same patients demonstrated sensitivities of 53.3 % (152/285, CI95 = [47.4–59.2]), 34.6 % (97/280, CI95 = [29.1–40.5]) and 40.1 % (116/289, CI95 = [34.4–46]), respectively. The antibody detection rates obtained in plasma samples by IPC ELISAs were significantly higher than those obtained in saliva and urine specimens by RDTs and IPC ELISAs (McNemar *p*-value ≤0.001 for all parameters).

For each of the three different antibody isotypes, there was a direct relationship between the level of antibodies in the plasma samples and the percentage of positive urine and saliva samples tested by IPC ELISAs and RDTs (Table 3). The higher the level of antibodies was in the plasma sample, the higher the probability was for the corresponding urine or saliva specimen to test positive.

**Table 3:** Detection rate of antibodies in urine and saliva samples

		Urine		Saliva	
		IPC ELISA	RDT	IPC ELISA	RDT
IgG	Weakly positive plasma	48.1 % (13/27)	29.6 % (8/27)	48.1 % (13/27)	29.6 % (8/27)
	Averagely positive plasma	50 % (41/82)	36.6 % (30/82)	74.4 % (61/82)	48.8 % (40/82)
	Highly positive plasma	72.1 % (31/43)	62.8 % (27/43)	93 % (40/43)	67.4 % (29/43)
	*p*-value ^a^	0.041	0.006	<0.001	0.008
IgM	Weakly positive plasma	0 % (0/25)	0 % (0/25)	40 % (10/25)	8 % (2/25)
	Averagely positive plasma	0 % (0/54)	0 % (0/54)	83.3 % (45/54)	22.2 % (12/54)
	Highly positive plasma	0 % (0/18)	0 % (0/18)	100 % (18/18)	44.4 % (8/18)
	*p*-value ^a^	1	1	<0.001	0.019
IgA	Weakly positive plasma	18.2 % (6/33)	0 % (0/33)	12.1 % (4/33)	0 % (0/33)
	Averagely positive plasma	50 % (31/62)	27.4 % (17/62)	62.3 % (38/61) ^b^	13.1 % (8/61) ^b^
	Highly positive plasma	100 % (21/21)	71.4 % (15/21)	81 % (17/21)	23.8 % (5/21)
	*p*-value ^a^	<0.001	<0.001	<0.001	0.021

The detection rates of antibodies in urine and saliva samples are presented according to the level of the immunoglobulin in the corresponding plasma samples

^a^ Comparison of antibodies positive rate in urine or saliva from patients whose corresponding plasma tested weakly, averagely or highly positive for the same biomarker

^b^ One invalid result

The IgG saliva-based tests performed better with samples collected between DAOF 8–43. The RDT reached a maximum sensitivity of approximately 60 % (61.5 % at DAOF 8–14; 59.6 % at DAOF 15–28; 57.1 % at DAOF 29–43) and the IPC ELISA a sensitivity of 78 % (79.5 % at DAOF 8–14; 78.9 % at DAOF 15–28; 75.7 % at DAOF 29–43) (Table 4). A total of 85 % of the plasma samples collected between DAOF 8–43 tested positive. Three months after the onset of fever, the sensitivity dropped to 55.9 % with the saliva-based ELISA and 8.8 % with the saliva-based RDT, whereas IgG were detected in 66.7 % of the corresponding plasma specimens (Table 4). The sensitivity of the IPC and RDT IgG urine-based tests peaked at DAOF 8–14 (ELISA: 71.8 %, RDT: 62.8 %). Then, the sensitivity of the ELISA and the rapid test decreased gradually. At DAOF 90–103, only 12.1 and 3 % of the urine samples still tested positive by ELISA and RDT, respectively (Table 4).

**Table 4:** Sensitivity of the different diagnostic tools for IgG, IgM and IgA detection

		DAOF (Day after onset of fever)						
		1–3	4–7	8–14	15–28	29–43	90–103	Total
Plasma ELISA	IgG	3.8 % (2/53)	48.2 % (54/112)	85.7 % (36/42)	NA	84.4 % (38/45)	66.7 % (22/33)	53.3 % (152/285)
	IgM	3.9 % (2/51)	49.1 % (55/112)	81 % (34/42)	NA	13.6 % (6/44)	0 % (0/31)	34.6 % (97/280)
	IgG or IgM	8.2 % (4/49)	64 % (71/111)	100 % (42/42)	NA	82.9 % (34/41)	78.6 % (22/28)	63.8 % (173/271)
	IgA	3.8 % (2/53)	47 % (54/115)	90.5 % (38/42)	NA	40.4 % (19/47)	9.4 % (3/32)	40.1 % (116/289)
Saliva ELISA	IgG	5.4 % (3/56)	38.2 % (86/225)	79.5 % (62/78)	78.9 % (45/57)	75.7 % (53/70)	55.9 % (19/34)	51.5 % (268/520)
	IgM	3.6 % (2/56)	44.4 % (100/225)	69.2 % (54/78)	35.1 % (20/57)	7.1 % (5/70)	0 % (0/34)	34.8 % (181/520)
	IgG or IgM	8.9 % (5/56)	56.9 % (128/225)	92.3 % (72/78)	94.7 % (54/57)	77.1 % (54/70)	55.9 % (19/34)	63.8 % (332/520)
	IgA	0 % (0/57)	24.8 % (55/222)	64.4 % (49/76)	18.2 % (10/55)	5.4 % (4/74)	0 % (0/36)	22.7 % (118/520)
Saliva RDT	IgG	0 % (0/56)	24.9 % (56/225)	61.5 % (48/78)	59.6 % (34/57)	57.1 % (40/70)	8.8 % (3/34)	34.8 % (181/520)
	IgM	0 % (0/56)	15.6 % (35/225)	24.4 % (19/78)	3.5 % (2/57)	1.4 % (1/70)	0 % (0/34)	11 % (57/520)
	IgG or IgM	0 % (0/56)	32 % (72/225)	70.5 % (55/78)	61.4 % (35/57)	57.1 % (40/70)	8.8 % (3/34)	39.4 % (205/520)
	IgA	1.8 % (1/57)	8.1 % (18/222)	17.1 % (13/76)	0 % (0/55)	0 % (0/74)	0 % (0/36)	6.2 % (32/520)
Urine ELISA	IgG	3.3 % (2/61)	41.3 % (93/225)	71.8 % (56/78)	58.5 % (31/53)	34.3 % (24/70)	12.1 % (4/33)	40.4 % (210/520)
	IgA	0 % (0/61)	25.7 % (58/226)	67.1 % (53/79)	50.9 % (27/53)	5.7 % (4/70)	0 % (0/33)	27.2 % (142/522)
Urine RDT	IgG	0 % (0/61)	25.8 % (58/225)	62.8 % (49/78)	45.3 % (24/53)	18.6 % (13/70)	3 % (1/33)	27.9 % (145/520)
	IgA	0 % (0/61)	11.5 % (26/226)	31.6 % (25/79)	9.4 % (5/53)	0 % (0/70)	0 % (0/33)	10.7 % (56/522)

The sensitivity of the different diagnostic tools for IgG, IgM and IgA detection in plasma, urine and saliva is presented according to the time of sampling after the onset of fever

*DAOF* Day After the Onset of Fever, *NA* no sample available

The second week after the beginning of the disease was the optimal time to detect IgM in both saliva and plasma samples. The IgM detection rates in saliva samples by ELISA and RDT reached a maximum of 69.2 and 24.4 %, respectively, whereas 81 % of the corresponding plasma samples tested positive by MAC-ELISA (Table 4).

All IgA tests demonstrated a maximal sensitivity at DAOF 8–14 (saliva: 64.4 % with ELISA and 17.1 % with RDT; urine: 67.1 % with ELISA and 31.6 % with RDT; plasma ELISA: 90.5 %). No positive results were obtained with RDTs at DAOF 29–43 whereas 5.4 % of the saliva, 5.7 % of the urine and 40.4 % of the plasma samples tested positive by IPC ELISA. Three months after the onset of fever, anti-DENV IgA were not detected by saliva- and urine-based tests but 9.4 % of the plasma samples still tested positive (Table 4).

Regardless of the diagnostic test, ELISA or RDT, and the biological fluid tested, the detection rates of anti-DENV IgG and IgA isotypes were higher in samples collected from patients experiencing a secondary infection (Table 5). With IPC and RDT tests designed for IgM detection in saliva and in plasma, no significant differences were observed between primary and secondary infections (Table 5).

**Table 5:** Sensitivity of the different tools for IgG, IgM and IgA detection

		Plasma	Saliva		Urine	
		IPC ELISA	IPC ELISA	RDT	IPC ELISA	RDT
IgG	Primary	16.1 % (13/81)	13.2 % (19/144)	8.3 % (12/144)	5.6 % (8/143)	1.4 % (2/143)
	Secondary	72.9 % (124/170)	68 % (208/306)	48 % (147/306)	56.8 % (171/301)	39.2 % (118/301)
	*p*-value ^a^	<0.001	<0.001	<0.001	<0.001	<0.001
IgM	Primary	31.2 % (24/77)	33.3 % (48/144)	11.1 % (16/144)	0 % (0/143)	0 % (0/143)
	Secondary	37.1 % (62/167)	34 % (104/306)	7.5 % (23/306)	0 % (0/301)	0 % (0/301)
	*p*-value ^a^	0.365	0.891	0.206	1	1
IgA	Primary	12.7 % (10/79)	2.9 % (4/140)	2.1 % (3/140)	4.2 % (6/143)	4.2 % (6/143)
	Secondary	53.2 % (91/171)	30.5 % (92/302)	6.6 % (20/302)	38.5 % (116/301)	14.3 % (43/301)
	*p*-value ^a^	<0.001	<0.001	0.049	<0.001	0.002

The sensitivity of the different tools for IgG, IgM and IgA detection in plasma, urine and saliva is presented according to the patients’ immune status

^a^ Comparison of sensitivity between primary and secondary infection

The antibody RDT was designed to concomitantly detect anti-DENV IgM and IgG. The combination of IgM and IgG results allowed an increase of the overall diagnostic sensitivity in saliva from 34.8 % with the IgG results alone to 39.4 % when both IgG and IgM were tested. This improvement in sensitivity was best for samples collected at DAOF 8–14 (9 % increase) and DAOF 4–7 (7.1 % increase). In comparison, 63.8 % of the plasma samples tested positive for dengue when IPC MAC-ELISA and IPC IgG indirect ELISA results were combined (Table 4).

## Discussion

The epidemiology of dengue is very dynamic both in terms of geographical spread and intensity. Transmission is emerging or reemerging in areas where the disease was absent and it is also intensifying in some regions where dengue was already endemic, with both an increase in the number of cases and an increase in severity of the disease [13]. This dramatic increase in the global burden of dengue has lead to the willingness to develop easy tools for the disease diagnosis, to help in the early detection of new epidemics and assist physicians to provide accurate treatment and management of patients as early as possible. The ability to rapidly confirm acute dengue infection for patients presenting to a clinical setting could also avoid the unnecessary use of antibiotics and other drugs.

Saliva and urine specimens provide interesting advances for dengue diagnosis as their collection is non-invasive and thus well accepted by patients. In addition, it does not require medically-trained staff and the samples are easy to process as it does not require on-site laboratory for centrifugation. In a previous study, we demonstrated that urine and saliva samples were interesting alternatives to venous blood specimens for dengue diagnosis in all situations when blood collection was difficult [11]. In this recent study, in-house ELISAs for NS1 antigen and anti-DENV antibodies detection were developed to assess the value of urine and saliva specimens for dengue diagnosis. We showed that antibody detection in saliva and urine was useful for instances such as outbreak investigations or in young children, in addition to the all situations when blood could not be easily collected (e.g., lack of phlebotomist, refusal of procedure, etc.). Good salivary- and urinary-based RDTs could be valuable tools for dengue diagnosis as they combine easy sampling methods with rapid, equipment-free testing. Such kits could be used by nurses, at the patient’s bedside, in hospital settings but also by general practitioners in private clinics or by epidemiologists during outbreak investigations and field studies.

Some oral fluid RDTs have already been marketed for viral infection diagnosis. One immunochromatographic assay designed for anti-hepatitis C virus (HCV) antibody detection in saliva and at least two anti-human immunodeficiency virus (HIV) antibody detection kits have been commercialized. The anti-HCV antibodies device showed very good performances [14, 15] and seemed to be an interesting tool for field work with people at risk of HCV infection [16, 17]. The anti-HIV antibody oral fluid devices also demonstrated good performances but slightly lower than RDTs used for antibody detection in blood [18–20]. There is currently no rapid test designed for the diagnosis of viral infection in urine but good RDTs have been developed for bacterial antigen detection in urine, e.g., for *Legionella pneumophila* serogroup 1 and *Streptococcus pneumoniae* [21–23]. A rapid immunochromatographic test for *Plasmodium falciparum* HRP2 antigen detection in urine is currently in clinical development and a preliminary evaluation reported a sensitivity of 83.8 % compared to blood smear microscopy, with a lower sensitivity in urine from patients with a low parasitemia [24].

This study was the first evaluation of rapid diagnostic tests designed by the Standard Diagnostics company to detect NS1 antigen and anti-DENV IgG, IgM and IgA in urine and saliva specimens. The present study demonstrates differing performances of these kits in comparison to the in-house ELISAs that we previously developed. The RDT designed for the detection of NS1 in saliva lacked sensitivity and specificity compared to the in-house ELISA, resulting in a very low kappa coefficient (0.28). The results obtained by the NS1 RDT with urine specimens were close to those observed with our ELISA with sensitivities of 15.5 and 14.3 %, respectively, and a kappa coefficient of 0.88. The slightly better performance observed with the RDT in comparison to the ELISA can be explained by a reduced sensitivity of the IPC in-house ELISA for the detection of NS1 protein from serotype 2 dengue viruses. Indeed all of the nine urine samples that tested positive with the RDT but negative with IPC ELISA (Additional file 2a) were obtained from patients infected by DENV-2. The reduced specificity observed with the RDT in saliva specimens could be a consequence of a non-specific adhesion of the detector colloidal particles, which are referred to as conjugates, to the nitrocellulose membrane. This phenomenon has been described with saliva but not with other body fluids. Indeed, mucin and other proteinaceous and viscous substances present in high concentration in saliva are believed to adhere to the membrane and aggregate with the conjugates causing non-specific reaction [25].

The IgG RDT demonstrated a good agreement, defined by a kappa coefficient ≥ 0.61 [26], with the in-house IgG indirect ELISAs, in both saliva (kappa coefficient: 0.62) and urine (kappa coefficient: 0.71) specimens. IgM and IgA RDTs performed less well in both saliva and urine. The agreement of these tests with the corresponding IPC ELISAs was only fair (0.21 ≤ kappa coefficient < 0.41) to moderate (0.41 ≤ kappa coefficient < 0.61). Sensitivities of the RDT for IgM and IgA detection in saliva were lower than 50 % even in patients with high levels of the corresponding antibody isotype in the plasma specimens collected at the same time-points. IgM levels peak in the serum about two weeks after the onset of symptoms and then decline generally to undetectable levels over 2–3 months. Anti-dengue serum IgG is generally detectable at low titres at the end of the first week of illness, increasing slowly thereafter, with serum IgG still detectable after several months, and probably even for life. During a secondary dengue infection IgG is detectable at high levels, even in the acute phase, and persists for periods lasting from 10 months to life. Early convalescent stage IgM levels are significantly lower in secondary infections than in primary ones and may be undetectable in some cases, depending on the test used. Combining IgM and IgG test therefore offers the possibility to serologically detect a dengue infection during a larger window of time as well as during some secondary infections when IgM are not detectable. In addition, the comparison of IgM and IgG results distinguishes primary and secondary dengue infections [2]. As a result, diagnostic kits combining both anti-dengue IgM and IgG antibodies became increasingly popular. As previously described by Vasquez et al., anti-DENV IgM were not found in urine, either by in-house ELISA or by RDT [7]. The combination of IgG and IgM detection in the same device was thus not useful to test urine samples. In saliva specimens, the combination of IgM and IgG tests in the same device provided a slight improvement of the overall diagnostic sensitivity compared to the sensitivities of IgG and IgM tests taken separately (39.8 % for the combination, 35.2 % for IgG alone and 10.8 % for IgM tested separately). Urine and saliva are biological fluids that contain very low levels of antibodies compared to blood [27, 28]. It was estimated that the IgA, IgG and IgM levels in saliva specimens were approximately 1/10, 1/800 and 1/400 of those measured in serum [27]. RDTs designed by SD company only required 5 μl of saliva and 10 μl of urine. These volumes ensure correct performances of the kits for the detection of anti-DENV antibodies in blood [29, 30] but could be sub-optimal for the detection of antibodies present in lower concentrations in other body fluids. Indeed, Zhang et al. developed a rapid test for the detection of anti-DENV IgG in saliva but this assay required a volume of 100 μl [25].

The high viscosity of some saliva specimens was a interfering with the migration of the sample on the strip test. Invalid results with RDTs were obtained because of the non-appearance of the control line. The use of a dilution buffer enabling to reduce the saliva viscosity could eventually prevent these migration issues as even a freeze–thaw cycle that in theory should break down the mucopolysaccharides responsible of the viscosity did not provide any improvement.

This study has several limitations. Saliva and urine samples were frozen for several months at −80 °C before the evaluation and it is not known if this process could have altered the stability of the NS1 antigen or the antibodies. Additionally, the performances of these diagnostic kits were not evaluated for all four dengue virus serotypes as this study was conducted using well-characterized and sequential clinical samples prospectively collected during a DENV-1 epidemic, when the DENV-2 and DENV-4 were circulating at lower level and no DENV-3 was detected. Specificity results should also be interpreted with caution as they were generated from a limited number of available negative controls. Moreover we used only samples from febrile, non-dengue patients to evaluate the specificity. Non-specific binding with the saliva RDTs might have been more pronounced in these samples than it would have been with samples from healthy individuals.

## Conclusions

Although urine and oral fluid-based rapid diagnostic tests offer an attractive apparent alternative option to blood for dengue diagnosis, this evaluation suggests that this first series of diagnostic tools developed by a commercial company really need further improvement especially in a context where the body fluids explored are already known to perform less well compared to blood specimens for the diagnosis of dengue [11]. As for any other diagnostic test evaluation, additional independent study would be beneficial in order to provide a better overview of the performances of these kits in different settings and epidemic situations.

### Ethics and consent to participate

The study was approved by the National Cambodian Ethics Committee for health research (authorization NECHR 28). A patient’s enrollment was subject to obtaining written consent signed by the participant or by a legal representative for participants under 16 years of age.

### Consent to publish

Not applicable.

### Availability of data and materials

All data can be made available upon request to the corresponding author.

## Additional files

Additional file 1: Characteristics of the rapid diagnostic tests for NS1, anti-DENV IgG/IgM and anti-DENV IgA detection in saliva and urine according to the manufacturer’s instructions. (PDF 87 kb) Additional file 2: Concordance between RDTs and IPC ELISAs for NS1, anti-DENV IgG/IgM and anti-DENV IgA detection in saliva and urine. (PDF 29 kb)

## Abbreviations

DAOF: day after onset of fever
DENV: dengue virus
DENV−1,−2,−3,−4: dengue virus serotype 1–4
ELISA: enzyme-linked immunosorbent assay
IPC: Institut Pasteur in Cambodia
RDT: rapid diagnostic test
SD: standard diagnostics
