# Supplementary material for: Evaluation of the performances of six commercial kits designed for dengue NS1 and anti-dengue IgM, IgG and IgA detection in urine and saliva clinical specimens
Source: BMC Infect Dis. 2016 May 16;16:201. doi: 10.1186/s12879-016-1551-x (PMC4867535; doi:10.1186/s12879-016-1551-x)
Supplement: Additional file 1: — Characteristics of the rapid diagnostic tests for NS1, anti-DENV IgG/IgM and anti-DENV IgA detection in saliva and urine according to the manufacturer’s instructions. (PDF 87 kb) [file 12879_2016_1551_MOESM1_ESM.pdf]

Additional file 1. Characteristics of the rapid diagnostic tests for NS1, anti-DENV IgG/IgM and anti-DENV IgA detection in saliva and urine according to the manufacturer.

	**Test 1**	**Test 2**	**Test 3**	**Test 4**	**Test 5**	**Test 6**
**Marker**	NS1	IgM and IgG	IgA	NS1	IgM and IgG	IgA
**Biologic fluid**	Saliva	Saliva	Saliva	Urine	Urine	Urine
**Volume of sample**	100 µl	5 µl	5 µl	100 µl	10 µl	10 µl
**Volume of diluent**	no diluent	~ 120 µl	~ 120 µl	no diluent	~ 120 µl	~ 120 µl
**Reading time**	20 minutes	20 minutes	20 minutes	15 to 20 minutes	15 to 20 minutes	15 to 20 minutes
