# Supplementary material for: Evaluation of the performances of six commercial kits designed for dengue NS1 and anti-dengue IgM, IgG and IgA detection in urine and saliva clinical specimens
Source: BMC Infect Dis. 2016 May 16;16:201. doi: 10.1186/s12879-016-1551-x (PMC4867535; doi:10.1186/s12879-016-1551-x)
Supplement: Additional file 2: — Concordance between RDTs and IPC ELISAs for NS1, anti-DENV IgG/IgM and anti-DENV IgA detection in saliva and urine. (PDF 29 kb) [file 12879_2016_1551_MOESM2_ESM.pdf]

Additional file 2. Concordance between RDTs and IPC ELISAs for NS1, anti-DENV IgG/IgM and anti-DENV IgA detection in saliva and urine.

a. NS1 urine

		IPC ELISA		
		Negative	Positive	Total
	Negative	368	4	372
	Positive	9	59	68
	Total	377	63	440
	Agreement: 97.1%; κ coefficient: 0.88			

b. NS1 saliva

		IPC ELISA		
		Negative	Positive	Total
	Negative	186	44	230
	Positive	31	28	59
	Total	217	72	289
	Agreement: 74.0%; κ coefficient: 0.28			

c. IgG urine

		IPC ELISA		
		Negative	Positive	Total
	Negative	308	67	375
	Positive	2	143	145
	Total	310	210	520
	Agreement: 86.7%; κ coefficient: 0.71			

d. IgA urine

		IPC ELISA		
		Negative	Positive	Total
	Negative	374	92	466
	Positive	6	50	56
	Total	380	142	522
	Agreement: 81.2%; κ coefficient: 0.42			

e. IgG saliva

		IPC ELISA		
		Negative	Positive	Total
	Negative	246	93	339
	Positive	6	175	181
	Total	252	268	520
	Agreement: 81%; κ coefficient: 0.62			

f. IgM saliva

		IPC ELISA		
		Negative	Positive	Total
	Negative	338	125	463
	Positive	1	56	57
	Total	339	181	520
	Agreement: 75.8%; κ coefficient: 0.36			

g. IgA saliva

		IPC ELISA		
		Negative	Positive	Total
	Negative	399	89	488
	Positive	3	29	32
	Total	402	118	520
	Agreement: 82.3%; κ coefficient: 0.32			
